# The Influence of Removable Complete Denture on Pro‐Oxidant Antioxidant Balance and Redox‐Sensitive Inflammation Biomarker NF‐*ĸ*B in the Oral Cavity: An Interventional Follow‐Up Study

**DOI:** 10.1002/cre2.70007

**Published:** 2024-09-19

**Authors:** Mirjana Bošković, Dušan Sokolović, Saša Stanković, Ivan Ristić, Jordan Popović, Gordana Kocić

**Affiliations:** ^1^ Department of Prosthodontics, Medical Faculty University of Niš Nis Nis region Serbia; ^2^ Department of Biochemistry, Medical Faculty University of Niš Nis Nis region Serbia; ^3^ Department of Oral Microbiology and Immunology, Institute of Odontology The Sahlgrenska Academy at University of Gotheburg Goteborg Goteborg region Sweden

**Keywords:** carcinogenesis, chronic inflammation, denture, oxidative stress

## Abstract

**Objectives:**

Oxidative stress, an imbalance between the body's natural antioxidant defenses and the production of reactive oxygen species (ROS), can result in serious oral diseases, including oral cancer, periodontal diseases, and oral lichen planus, through the activation of the redox‐sensitive transcription factors and inflammation. The purpose of this study was to assess the potential effects of a removable complete denture on the levels of oxidative stress markers, such as lipid peroxidation (MDA), advanced oxidation protein products (AOPP), and catalase, and the quantitative expression of the redox‐sensitive transcription factor NF‐*κ*B p65 subunit.

**Materials and Methods:**

This interventional follow‐up study enrolled 40 participants of both sexes aged 28–78 years, with a median age of 56 years, where unstimulated saliva was collected before denture placement, immediately after the denture placement, and 24 h, 7 days, and 30 days after the denture placement. The most prominent ROS overproduction was reported on the seventh day (*p* < 0.05), followed by a significant fall in antioxidative defense.

**Results:**

The NF‐*κ*B p65 subunit, whose expression pattern was highest in the same time period on the seventh day, serves as a signaling molecule for redox imbalance due to ROS production. Over the next 30 days, its levels remained moderately increased compared to the basal value, which may influence pro‐inflammatory pathways and the integrity of oral tissue components. These alterations may be induced by the dentures, which can produce high pressures on the supporting tissues or by the synthetic materials used for producing the dentures.

**Conclusion:**

Our research may help to clarify the potential pathways by which oxidative stress and redox‐sensitive inflammatory mediators, as well as mechanical and chemical irritants, may serve as risk factors for premalignant lesions in the mouth. Further research on this topic is required to understand the molecular mechanisms behind the relationship between inflammation and oral premalignant lesions caused by mechanical and chemical irritation.

## 1 Introduction

Oxidative stress has been described as an imbalance between the body's endogenous antioxidant defenses and reactive oxygen species (ROS) production. It has been related to many pathologies, such as cardiovascular disease, neurodegenerative disorders, and cancer, and physiological aging process (Valko et al. 2007). The consequences are impaired functional activity, damage to essential cell components, membrane damage, and consequent cytolysis. The mechanism of inflammation in injured tissues can be linked to ROS production via the activation of the pleiotropic regulator of immune and inflammatory responses, nuclear factor kappa‐B (NF‐*κ*B) (Mitchell, Vargas, and Hoffmann 2016). Chronic trauma and chemical damage of oral mucosa may lead to epithelial tissue remodeling toward morphologically different pre‐cancerous lesions, such as epithelial dysplasia, epithelial hyperplasia, and finally to the development of oral cancer. The role of chronic inflammation may represent a cellular mechanism mediating in the pathogenesis of epithelial cancer of the oral cavity induced by chemical and mechanical trauma (Singhvi, Malik, and Chaturvedi 2017; Balkwill, Charles, and Mantovani 2005). Pro‐inflammatory transcriptional factor NF‐*κ*Β signaling pathway, particularly through its p65 subunit activation, represents a main downstream target of many redox‐active inflammatory mechanisms involved in cell differentiation and the early stages of epithelial carcinogenesis of the oral cavity (Kamperos et al. 2016; Nakayama et al. 2001; Ghosh et al. 2016; Mishra et al. 2006). The presence of chemical mediators and different pathogens may promote inflammation‐induced cancer by activating NF‐*κ*B (Shrihari et al. 2016). In the current literature, there are no data about the effect of mobile denture materials on the NF‐*κ*B signaling pathway of mucosa epithelial cells. A removable complete denture is a prosthodontic appliance used for the treatment of partial and total edentulism, usually in elderly individuals. Its main task is to restore the masticatory function and aesthetic appeal of one's dental arch without doing direct harm to the patient. Biocompatibility of materials, as well as the way removable complete denture is made, can have a very high impact on the tissues of the oral cavity, including mucosa, underlying bones and blood vessels. Mobile total dentures are usually made from acrylic. The main substance is methyl methacrylate that can have hemotoxic effects and can be a potential cause of oxidative stress (Nishimiya et al. 2015; Schweikl, Spagnuolo, and Schmalz 2006; Krifka et al. 2013; Alizadehgharib et al. 2020; Goldberg 2008). Possible alterations in oxidative homeostasis and chronic inflammation triggered by reactive species production after the placement of a removable complete denture may cause harm to the patient since the removable complete denture stays in the patient's oral cavity for a prolonged period of time. Currently, there is no published research overlooking the effect of the removable complete denture on oxidative status and inflammation markers. The markers of choice in the present study were malondialdehyde (MDA) and advanced oxidation protein products (AOPP) as markers of oxidative stress, activity of the antioxidative enzyme catalase, and NF‐*κ*B as the link between ROS production and pro‐inflammatory pathways.

Our hypothesis is that prolonged and excessive oxidative stress can cause inflammation by triggering the redox‐sensitive transcription factor NF‐*κ*B and further triggering the expression of pro‐inflammatory genes. Prolonged inflammation may cause proliferation, dysplastic aberration of epithelial cells, stimulation of oncogenic growth factors, and the formation of precancerous lesions.

The aim of our study was to evaluate if there was a significant shift of observed markers before the placement of the removable complete denture and over the next 30 days.

## 2 Materials and Methods

### 2.1 Participants

The single‐arm clinical trial, designed as an interventional follow‐up study, enrolled patients seeking treatment for edentulism at the Department of Prosthodontics of the Clinic of Dentistry in Niš.

All subjects underwent an obligatory clinical examination. The first examination was performed to make sure that the removable complete denture was the possible treatment of choice, according to the clinical findings and patients’ requests. For patients to be included as participants in our study, the protocol was designed to enable the study sample as representative as possible.

The sole requirements for inclusion were non‐smoking patients who had never smoked and did not have any illnesses or anomalies involving the tissues or organs of the mouth. The patients were as close to the ideal state for entire denture construction as clinically possible, with fully healthy alveolar ridges without substantial resorption and oral mucosa and organs for the conduct of our pilot study. The healthy oral mucosa in the mouth should be pink, smooth, and wet. Forty individuals, aged 28–78, with a median age of 56, made up the final group for saliva collection. The same doctor performed a thorough oral examination on a chosen group of patients.

Patients who underwent quick denture delivery and had detachable partial dentures as a therapy option for partial edentulism were excluded based on denture type. During the general medical examination, the following conditions were ruled out as potential exclusion criteria related to inflammation: any symptoms of acute inflammatory or other acute viral and bacterial illnesses, autoimmune diseases, diabetes mellitus, chronic systemic diseases, cardiovascular diseases (myocardial infarction and coronary heart disease), cerebrovascular diseases, deep vein thrombosis and pulmonary embolism, multiple comorbidities, frequent hospitalizations, shortness of breath, severe rheumatic pain, and obesity. It was also essential that the patients were not receiving any medical treatments at the moment or taking any drugs that altered salivary flow or composition.

To start the prosthodontic treatment, adequate preparation was done according to each case.

#### 2.1.1 Removable Complete Denture Production

All of the removable complete dentures were made identical by the same dental technician using the same material.SR Triplex Hot is a heat‐curing, PMMA‐based denture base material, consisting of powder (polymethyl methacrylate, catalyst, pigments) and liquid (methyl methacrylate stab., dimethacrylate). The powder and liquid were mixed in a ratio of 23.4 g of powder:10 mL of liquid. After that, the material needed to mature at room temperature (23°C/73°F) for about 10 min and then it stops being sticky. A sufficient amount of material was placed in one half of the flask and closed with the other half in a clamp under a pressure of 80 bar. Thermal polymerization begun in cold water, which was heated to 100°C and boiled for 45 min. After polymerization, the flask was cooled for about 30 min at room temperature and then placed in cold water until complete cooling. In the end, the removable complete denture was released from the flask and polished in the usual way. The denture was kept in water until it was handed over to the patient. All patients were instructed on how to take care of their removable complete dentures and oral cavities in the same way after the removable complete denture was placed.

#### 2.1.2 Saliva Sampling

In the follow‐up study, the unstimulated saliva was collected in five time intervals: before removable complete denture placement, immediately after the removable complete denture placement, and 24 h, 7 days, and 30 days after the removable complete denture placement. To prevent any circadian variation, a 2‐h tolerance threshold was set for saliva sampling.

We sampled unstimulated saliva from 40 patients by the method of Granger et al. (2007) each time between 8:30 and 9:30 am. Saliva was carefully routed through a plastic tube into Eppendorf tubes of 2.0 mL. Five samples from each patient were taken. The first sample was taken after the clinical examination and before the removable complete denture placement; therefore, it has been used as the control level. The second sample was taken after the placement of a removable complete denture, the third 24 h after, the fourth 7 days after, and the fifth 30 days after the insertion of the removable complete denture. All saliva samples were stored in an ice container maintaining the temperature of 4°C until centrifuged for 12 min at 3000 r/min while keeping the temperature unchanged. The supernatant was stored at −80°C until the analyses were performed. The maximum time of storage was 30 days.

#### 2.1.3 Determination of MDA

The intensity of lipid peroxidation in saliva supernatant samples was measured using the method of Ohkawa et al., based on the spectrophotometric determination of thiobarbituric acid (TBA) reactive product MDA at 532 nm on the ELISA microplate reader (Ohkawa, Ohishi, and Yagi 1979). The MDA concentration was expressed as nmol/mg of protein, by using the molecular extinction coefficient of MDA (1.56 × 10**^−5^** mol/cm).

#### 2.1.4 Determination of AOPP

The level of AOPP was determined in saliva samples using a spectrophotometric method with an ELISA microplate reader, where the concentration was calibrated with chloramine T as standard. In test wells, 200 μL of saliva, diluted 1:5 in PBS were placed on a 96‐well microtiter plate afterward 10 µL of 1.16 M potassium iodide (KI) was added, followed by the addition of 20 µL of acetic acid. In standard wells, 200 μL of chloramine T solution was added in different concentrations (0–100 μmol/L), 10 μL of 1.16 M potassium iodide, followed by 20 μL of acetic acid. The absorbance of the reaction mixture was measured at 340 nm against a blank containing 200 μL of PBS, 10 μL of potassium iodide, and 20 μL of acetic acid. The obtained AOPP concentrations were expressed as micromoles per litter of chloramine T equivalents. After measuring the protein concentration, AOPP was expressed as micromoles of chloramine T per liter per milligram of protein (Gradinaru et al. 2018).

#### 2.1.5 Determination of NF‐*k*B

All chemicals were purchased from Sigma, and the antibodies were purchased from Santa Cruz Biotechnology. NF‐*κ*B was determined, as previously described by Kocic et al. (2011, 2017). Briefly, the samples of saliva supernatant (10 μL) were immobilized on a flat‐bottomed solid 96‐well polystyrene microtiter plate, with two plates for each sample (serving as the test and the control plates). Afterward, bicarbonate buffer (100 mM, pH 9.6) was added. The NF‐*κ*B p65 present in saliva supernatant allowed to attach at 4°C overnight. Specific antigen–antibody binding was achieved by incubating the test plates with the corresponding primary antibody at 4°C for 24 h (anti‐NF‐*k*B p65 primary antibody p65 C‐20: sc‐372, epitope mapping at the C‐terminus of NF‐*k*B p65), which was diluted 1:1000 in PBS buffer/2.5% BSA. After washing, the detection assay was performed by the addition of a secondary FITC‐conjugated antibody (sc‐7972 FITC) in both, test and control plates, allowed to stay for 2 h in the dark. The excess secondary antibodies were washed, and the intensity of fluorescence was analyzed on a Victor Perkin Elmer‐Wallac multiplate reader. The fluorescence of the corresponding control plates was subtracted from the test plate values, and the quantity was expressed as the logarithm of fluorescence per quantity of cellular proteins.

#### 2.1.6 Determination of Catalase Activity

Catalase activity was determined using the spectrophotometric method by Goth (1991), based on the ability of catalase to break down the substrate (H_2_O_2_), with the enzymatic reaction, which has been stopped by the addition of ammonium molybdate, and the created yellow complex of H_2_O_2_ and molybdate has been measured at 405 nm against reagent blank. Enzyme activity was expressed in catalytic units per 1 g of protein (U/g protein).

Protein measurement in saliva was performed according to the Lowry method using a Folin phenol reagent (Lowry et al. 1951).

The values of the analyzed markers are determined pre‐treatment (prt), right after the treatment (rat), 24 h post‐treatment (24hpt), 7 days post‐treatment (7dpt), and 30 days post‐treatment (30dpt).

#### 2.1.7 Statistical Analysis

The analyzed variables were continuous and were reported as means with standard deviations and medians. The distribution of these variables was determined using the Shapiro–Wilk test. In case there was a normal distribution of the variables, the paired samples *t*‐test was used. If the distribution deviated from the normal distribution of the variables, the Wilcoxon signed‐ranks test was used. This statistical analysis was done using the program SPSS 15.0.

## 3 Results

**Figure 1:**
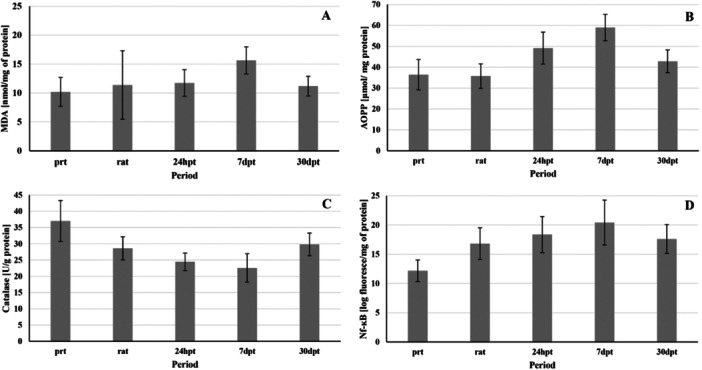
Clustered charts with a standard deviation of the (A) MDA, (B) AOPP, (C) catalase, and (D) NF‐*ĸ*B.

When observing the dynamic data shown in Tables 1, 2 and 2 and Figure 1, the peak of increase of pro‐oxidative markers MDA and AOPP has been recorded after 7 days. Catalase recorded its lowest value after 7 days (Table 3). MDA and NF‐*κ*B had the lowest values before the treatment (Tables 1, 4 and 4). AOPP had the lowest value right after the first treatment, although basal levels and levels right after the treatment did not differ significantly (Table 2). Levels of MDA, AOPP, and NF‐*κ*B p65 remained at higher levels during the whole follow‐up period of 30 days (Figure 1). Catalase activity showed the highest value before the treatment, whereas the most significant fall was registered after 7 days, gradually increased over a whole follow‐up period of 30 days, but it was still lower than basal values on the 30th day, indicating that the intervention effect persisted over time (Table 3 and Figure 1). The shift in levels of all markers was statistically significant, and a detailed comparison with *p* values is given in Tables 1–4.

**Table 1:** MDA over 30 days. Data are given as mean ± standard deviation (median) with the statistical significance.

	Pre‐treatment	Right after the treatment	24 h post‐treatment	7 days post‐treatment	30 days post‐treatment
MDA (nmol/mg of protein)	10.19 ± 2.49 (11.04)	11.38 ± 5.92 (10.59)	11.73 ± 2.30 (11.85)	15.63 ± 2.34 (15.33)	11.19 ± 1.70 (10.88)
Statistical significance				*p* < 0.001 vs. prt and rat	*p* < 0.05 vs. prt

**Table 2:** AOPP over 30 days. Data are given as mean ± standard deviation (median) with the statistical significance.

	Pre‐treatment	Right after the treatment	24 h post‐treatment	7 days post‐treatment	30 days post‐treatment
AOPP (μmol/mg of protein)	36.42 ± 7.2 (35.77)	35.74 ± 5.84 (34.08)	49.15 ± 7.65 (49.47)	59.00 ± 6.31 (62.05)	42.84 ± 5.44 (44.07)
Statistical significance			*p* < 0.005 vs. prt	*p* < 0.005 vs. prt and 30dpt	*p* < 0.05 vs. prt

**Table 3:** Catalase over 30 days. Data are given as mean ± standard deviation (median) with the statistical significance.

	Pre‐treatment	Right after the treatment	24 h post‐treatment	7 days post‐treatment	30 days post‐treatment
Catalase (U/g of protein)	37.02 ± 6.30 (37.30)	28.60 ± 3.57 (27.89)	24.46 ± 2.69 (24.56)	22.57 ± 4.37 (23.18)	29.80 ± 3.50 (28.76)
Statistical significance	*p* < 0.005 vs. rat, 24hpt and 7dpt *p* < 0.05 vs. 30dpt				*p* < 0.005 vs. 7dpt

**Table 4:** NF‐*ĸ*B p65 unit over 30 days. Data are given as mean ± standard deviation (median) with the statistical significance.

	Pre‐treatment	Right after the treatment	24 h post‐treatment	7 days post‐treatment	30 days post‐treatment
NF‐*ĸ*B (log fluoresce/mg of protein)	12.16 ± 1.85 (12.14)	16.80 ± 2.72 (18.00)	18.36 ± 3.09 (17.23)	20.41 ± 3.84 (20.27)	17.60 ± 2.48 (17.79)
Statistical significance		*p* < 0.001 vs. prt	*p* < 0.001 vs. prt	*p* < 0.001 vs. prt	*p* < 0.001 vs. prt

## 4 Discussion

In our interventional follow‐up study, which enrolled 40 patients, the unstimulated saliva was collected in five time intervals: before the removable complete denture placement, immediately after the removable complete denture placement, and 24 h, 7 days, and 30 days after the removable complete denture placement. We investigated markers of oxidative stress (MDA and AOPP levels), antioxidative defense (catalase activity), and redox‐sensitive inflammation factor (NF‐*κ*B p65 unit) in the saliva of the patients who got the removable complete denture for the first time. The data showed that the presence of the removable complete denture induced a marked shift of the measured markers in terms of increased MDA, AOPP level, and NF‐*κ*B and decreased catalase activity.

The similarity of saliva to plasma makes it a biological fluid suitable for the diagnostics of many diseases and for determining levels of different disease markers. It represents an environment where ROS may play an important role in redox‐dependent signaling and may induce oxidative damage to cellular and collagen structures of the oral cavity with serious pathophysiological consequences (Wang et al. 2015; Battino et al. 2002; Iannitti, Rottigni, and Palmieri 2012; Lee and Wong 2009). The time of sampling was always between 8:30 and 9:30 a.m. to prevent the influence of hormonal shifts in circadian rhythm that could lead to changes in saliva composition, which would cause the production of uncomparable samples (Nam et al. 2019). Patients who had systemic or oral diseases were excluded from the study to prevent the influence of these diseases on levels of measured markers during the 30 days after denture placement.

From the perspective of overall health, having appropriate dental compensation is undoubtedly advantageous for the patient, and this research should not alter that belief. Given that the benefits always outweigh the risks, this is presumably the reason why not much information is available about it. Even though these features of the detrimental consequences of a removable complete denture have not been extensively studied, it is nonetheless important to highlight potential side effects.

Oxidative stress can be caused by various factors including chemical species, radiation injury, hypoxia, ischemia, cellular aging, and tissue injury caused by inflammatory cells.

We hypothesize that, in addition to methyl methacrylate already indicated, the cause of oxidative stress may be the pressure exerted by the removable complete denture on the mucosa, which is damaging small blood vessels.

This mechanism is related to disturbed mitochondrial respiration due to hypoxia. Mitochondrial respiration is a normal metabolic function, although this reaction is not perfect. When the supply of oxygen is reduced, like in ischemia and lower perfusion of the tissue, larger amounts of superoxide, which is a free radical, are generated, among other reactive species (Lushchak 2014).

Oxidative stress as the metabolic disturbance has been linked over the years of research with inflammation, carcinogenesis, and other pathologies through the induction of complex chain reactions that lead to the transformation of cells (Fang, Seki, and Maeda 2009; Bhattacharyya et al. 2014; Visconti and Grieco 2009).

The estimation of MDA in saliva was chosen based on the literature data indicating that MDA is the most frequently measured stable product of polyunsaturated fatty acid peroxidation and increases in an oxidative stress‐dependent manner; the estimation of AOPP reflects protein oxidative modification, collagen oxidative damage due to covalent cross‐linking; the estimation of catalase was chosen because of the role of this enzyme as the first defense line against ROS. We suppose that these alterations are local and affect tissues of the oral cavity, based on evidence suggesting that shifts in oxidative stress and related markers are present in saliva. However, what poses a threat in the case where there is a prosthodontic appliance over a prolonged period of time is the state of chronic oxidative stress, which is said to induce multiple pathologic alterations in cells due to accumulated oxidative transformations of proteins and lipids (Zukowski, Maciejczyk, and Waszkiel 2018). Furthermore, chronic oxidative stress could lead to chronic inflammation. Results that we obtained suggest indeed that observed alterations could be chronic since the levels of markers were not the same as the ones in the control group even after 30 days. The mechanism of inflammation in injured tissue may occur via the activation of the pleiotropic regulator of the immune and inflammatory response, heterodimeric transcription factor NF‐*κ*B. The typical form of NF‐*κ*B is a heterodimer composed of p65/RelA and p50 subunits. This transcription factor could be activated by low and untraceable levels of ROS, which represent the role of oxidative stress in the activation of cellular proliferation and inflammatory alterations (Reuter et al. 2010). The role of NF‐*κ*B seems to be crucial for the tooth germ epithelium development and differentiation, bone morphogenesis, mucosal proliferation, and differentiation. Enhanced expression of NF‐*κ*B was documented in about 60 chronic inflammatory diseases and states, known as “NF‐*κ*B diseases,” whereas constitutive activation of NF‐*κ*B was documented also in human cancer cells, pointing its high sensitivity to different stimuli that can activate it, as well as several biological responses it may control (Adler et al. 2007; Amiri and Richmond 2005; Kunnumakkara et al. 2020).

The activation of NF‐*κ*B may be induced by the action of inflammatory cytokines such as TNF‐α and IL‐1, T‐cell activation signals, growth factors, and different stress inducers. Activated NF‐*κ*B is followed by phosphorylation of IκB and p65 unit nuclear translocation (Karin and Ben‐Neriah 2000).

One of the most significant problems is damage to the tooth tissue's cellular and membrane components (Jobin and Sartor 2000; Nichols et al. 2001).

Data from the literature indicate that thrombosis, systemic disorders, and cardiovascular diseases that are followed by “sterile inflammation” can cause retrograde inflammatory processes in the gingiva and alterations in the oral mucosa. Even if our study did not include individuals with these conditions, conversely, systemic diseases such as atherosclerosis and thrombotic problems are associated with periodontitis and generated inflammatory reactions (Isomura and Morita 2006; Shimizu et al. 2009).

The increased inflammatory response can trigger the extracellular flux of free radicals mainly by activated leukocytes.

The expression of NF‐*κ*B was enhanced in oral mucositis and periodontitis. The possible connection between periodontitis and ROS production may occur via the activation of NF‐*κ*B, which can induce tissue inflammation and further impairment of the tooth structure.

Activated NF‐*κ*B may trigger mechanisms of inflammatory cytokine secretion. Possible inhibition of NF‐*κ*B may prevent periodontitis and may promote wound healing in bone defects via inhibition of osteoclastic resorption, followed by the active loss of tooth structure.

The literature data discussing removable complete dentures from this point of view are obscure; however, several researchers have reported the shift in levels of oxidative stress markers in saliva and cervical gingival fluid with common oral pathologies such as periodontitis, oral lichen planus, temporomandibular joint disorders, and smoking (Akalin et al. 2007; Borges et al. 2007; Zhang, 2002; Rodríguez De Sotillo et al. 2011; Guentsch et al. 2008). Trivedi et al. showed altered antioxidative defense and higher levels of oxidative stress markers in saliva and blood of the patients with periodontitis and diabetes (Trivedi et al. 2014). Vlkova et al. suggested that markers of lipid peroxidation and carbonyl stress were increased in patients with oral premalignant lesions (Vlková et al. 2012). Decreased antioxidant status potentially due to decreased expression of antioxidant enzymes might be responsible for these findings.

All of the dentures in our research were removable complete dentures made for patients with complete edentulism. Removable partial dentures as the option for the treatment of patients with partial edentulism were not included in our study. Clinical experience shows that patients using removable partial dentures have very similar complaints as the patients who use removable complete dentures. Also, it is known that the pressure from the denture in both cases transfers to the mucosa and alveolar ridges in the same way with both removable complete and removable partial dentures. For these reasons, we believe that both complete and partial dentures might cause similar alterations in levels of these markers, which could be the topic of further research since there is a documented risk of periodontal disease and tooth structure alterations connected to the state of oxidative stress (Van Dyke and Serhan 2003; Akalin, Toklu, and Renda 2005; Biju et al. 2014; Corbi et al. 2014).

The limitation of this follow‐up study is that it has been designed to examine the degree to which effects are seen shortly after the intervention, but we need long‐term research to examine if the mentioned alterations persist over a longer time. Further research is required for a more in‐depth analysis of the influence that removable complete dentures and materials used for denture production have on the biology of the oral tissues. Future research could focus on comparing measured levels with a higher number of homogenous groups of participants. Determination of other oxidative stress markers might also provide an additional explanation of the underlying mechanism of redox signaling alterations. Nevertheless, the systemic effect that may appear would still apply the levels of ROS markers in serum to be valuable for comparison with saliva in our future research. In the present case, levels of markers were decreasing with time. However, we believe that 1 month of disturbed oxidative status is enough to suggest that these alterations are chronic.

The purpose of identifying the specified time points is to demonstrate that, although being an acute reaction, the oxidative stress that occurs here is significantly mitigated by compensatory mechanisms. This follow‐up serves as evidence that the 30‐day should be a possible check‐point when a reduction can be seen. This study's key finding is that this is the period during which oxidative stress parameters should be watched closely. If they remain noticeably out of the ordinary, this could be a sign that action needs to be taken to address the underlying causes of a protracted loss of pro‐antioxidant balance.

## 5 Conclusions

This study provided evidence that the most prominent ROS overproduction, estimated by the level of MDA and AOPP, appeared 7 days after the removable complete denture placement, followed by a significant fall in antioxidative defense enzyme catalase. The redox imbalance, due to excessive ROS production, promoted signaling molecules within redox‐sensitive pathways, such as the NF‐*κ*B p65 subunit, which showed the highest expression level on the seventh day. Over the next 30 days, their levels remained moderately affected compared to the basal value which may influence pro‐inflammatory pathways and oral tissue component integrity. Further research on this topic is required to determine the molecular mechanism underlying the link between inflammation and oral premalignant lesions due to mechanical and chemical irritation related to the mobile denture.

## Author Contributions

**Mirjana Bošković:** review and editing (equal), conceptualization (lead), writing–original draft (supporting), methodology (equal), formal analysis (supporting), supervision (lead), data curation (lead), visualization (lead). **Dušan Sokolović:** review and editing (equal), conceptualization (supporting), formal analysis (supporting), methodology (equal), supervision (equal). **Saša Stanković:** review and editing (equal), validation (lead), conceptualization (supporting), project administration (lead), resources (lead). **Ivan Ristić**. review and editing (equal), data curation (supporting), visualization (equal). **Jordan Popović:** review and editing (lead), writing–original draft (lead), formal analysis (equal), visualization (supporting), methodology (supporting). **Gordana Kocić:** conceptualization (equal), validation (lead), supervision (equal), writing–original draft (supporting), writing–review and editing (equal).

## Conflicts of Interest

The authors declare no conflicts of interest.

## Data Availability

The data that support the findings of this study are available from the corresponding author upon reasonable request.
